# Assessing the Quality of Antenatal Care in King Abdulaziz University Hospital: A Retrospective Study

**DOI:** 10.7759/cureus.37150

**Published:** 2023-04-05

**Authors:** Ayman A Bukhari, Dana N Alhibshi, Hanan M Alsayyad, Rozan I Altaifi, Kholoud A Althakafi

**Affiliations:** 1 Obstetrics and Gynecology, King Abdulaziz University, Jeddah, SAU; 2 Medicine, King Abdulaziz University, Jeddah, SAU

**Keywords:** missed appointments, maternal health, virtual clinics, ultrasounds, saudi arabia, covid-19, antenatal care

## Abstract

Background: Antenatal care is vital for pregnant women and fetuses. However, the coronavirus disease 2019 (COVID-19) pandemic has hindered access to care worldwide, resulting in missed appointments. Therefore, assessing the quality of antenatal care during the pandemic is crucial. This study evaluated the care provided at King Abdulaziz University Hospital in Saudi Arabia and suggested areas for improvement.

Methods: This retrospective medical records review involved 400 pregnant patients who received antenatal care at King Abdulaziz University Hospital in the past two years. A checklist was used to collect patient data, including demographics, antenatal care visits, ultrasounds, gestational age at first visit and ultrasound, prior cesarean section and preterm delivery, and virtual clinic attendance during the COVID-19 pandemic. Statistical analyses were performed using SPSS version 25 (Armonk, NY: IBM Corp.).

Results: The sample had a mean age of 30±6 years, and most participants (87.8%) were Saudi women. Over half of the participants did not attend any antenatal follow-up visits, and the majority had only one ultrasound. Only a small proportion of mothers attended virtual clinics during the pandemic. Having a prior cesarean section and a parity of 1-3 were positively associated with ultrasound attendance, while prior preterm delivery was positively associated with antenatal visits and virtual clinic attendance.

Conclusion: This study highlighted the importance of improving antenatal care quality at King Abdulaziz University Hospital, especially during COVID-19. To achieve this, strategies such as increasing visits, ultrasound attendance, and virtual clinic access should be considered. By implementing these recommendations, the hospital can enhance care and promote maternal and fetal health.

## Introduction

Antenatal care is an essential aspect of maternal healthcare that focuses on providing comprehensive services to ensure the well being of both the mother and the developing fetus [1]. The World Health Organization recommends a minimum of eight antenatal care visits for uncomplicated pregnancies, with additional visits for high-risk pregnancies. These visits involve various interventions, such as screening for infections, monitoring fetal growth and well being, providing education on healthy behaviors and pregnancy-related complications, and offering immunizations and nutritional supplements as appropriate [2,3]. These interventions are crucial in ensuring that women have a healthy pregnancy and a positive birth outcome [4].

The COVID-19 pandemic has highlighted the importance of antenatal care services. Pregnant women are at an increased risk of adverse outcomes due to the potential risk of exposure to the virus. The pandemic has disrupted antenatal care services worldwide, leading to reduced access to care and missed appointments [5,6]. The measures implemented to curb the spread of COVID-19 have made it challenging for pregnant women to attend antenatal care visits, leading to delays in seeking care and increasing the risk of adverse outcomes [7,8].

In Saudi Arabia, maternal healthcare has been prioritized, and the government has invested significantly in expanding antenatal care services and improving maternal and child health outcomes [9]. However, there is limited research on the quality of antenatal care services in the country and adherence to guidelines. Therefore, it is essential to evaluate the quality of antenatal care services in Saudi Arabia to ensure that pregnant women receive adequate and appropriate care, particularly during the COVID-19 pandemic.

To address this gap in knowledge, this study aimed to evaluate the quality of antenatal care services at King Abdulaziz University Hospital - one of the largest tertiary care hospitals in Saudi Arabia. The study will also focus on reviewing data for pregnant women during the COVID-19 pandemic and recommend strategies to improve the quality of antenatal care services at the hospital. The findings from this study are expected to contribute to improving maternal healthcare in Saudi Arabia and provide insights into the challenges faced by pregnant women.

## Materials and methods

Study design

This retrospective cohort study aimed to investigate the impact of the COVID-19 pandemic on antenatal care and delivery outcomes of pregnant patients. The study was conducted at the Department of Obstetrics and Gynecology, King Abdulaziz University Hospital, Jeddah, Saudi Arabia, from January 2020 to December 2021.

Study population

The study population consisted of 400 pregnant patients who had received antenatal care at King Abdulaziz University Hospital during the study period. Patients were included if they had attended at least one antenatal visit and had a documented delivery outcome. Patients with incomplete medical records or who had received antenatal care at another facility were excluded from the study.

Data collection

A standardized checklist was developed to collect the following information for each patient: mother's age, nationality, number of antenatal care visits, gravidity, parity, number of ultrasounds performed, gestational age at the first visit, date of the first ultrasound, gestational age at the first ultrasound, post-COVID-19 attendance at virtual clinics, number of virtual clinics attended, prior cesarean section, and prior preterm delivery.

Data were collected from electronic medical records by trained research assistants who were blinded to the study hypothesis. The collected data were entered into a secure electronic database. A random sample of 10% of the records was reviewed by a second research assistant to ensure the accuracy of data entry.

Statistical analysis

The collected data were coded, tabulated, and analyzed using SPSS version 25 (Armonk, NY: IBM Corp.). Descriptive statistics were used to summarize the data, and qualitative data were expressed as numbers and percentages. To test the relationship between variables, the chi-squared test (χ2) was applied for categorical data, and the results were reported as a p-value. For continuous data, the mean and standard deviation (mean ± SD) were calculated, and the nonparametric Kruskal‒Wallis test was used to compare differences between groups, with a p-value <0.05 considered statistically significant.

Ethical considerations

This study was approved by the Institutional Review Board at King Abdulaziz University Hospital. Informed consent was waived as the study was retrospective and the data were anonymized to protect patient confidentiality. The study was conducted in accordance with the Declaration of Helsinki and all applicable ethical guidelines.

## Results

Demographic characteristics

A total of 400 pregnant women were included in this study, with a mean age of 30±6 years. The majority of the sample (87.8%) were Saudi women, and the mean weight was 73.3±14.8. Most of the participants had no prior cesarean section (67.2%), and only a small minority (8.5%) had a history of preterm delivery (Table 1).

**Table 1:** Demographic characteristics of mothers.

Parameter		Frequency	Percent
Mother's age (years)	18-26	114	28.4%
	27-34	189	47.0%
	35-44	99	24.6%
	Mean ± SD (range)	30±6 (18-44)	
Nationality	Non-Saudi	49	12.2%
	Saudi	353	87.8%
Mother's weight	Mean ± SD (range)	73.3±14.8 (45.0-131)	
Prior cesarean section	No	270	67.2%
	Not applicable	20	5.0%
	Yes	112	27.9%
Prior preterm	No	319	79.4%
	Not applicable	49	12.2%
	Yes	34	8.5%

In terms of medical complications during pregnancy, it was found that 13.5% of the study population experienced complications, with 5% of these cases experiencing gestational diabetes, 3% with hypertension, and 5.5% with other complications.

Antenatal care

More than half of the participants (53.5%) did not attend any antenatal follow-up visits, and 49% had a gravidity of 1-3, with a similar proportion (57.5%) having a parity of 1-3. The majority of women (58.5%) had only one ultrasound, and almost half (49.3%) had one to two antenatal visits. The mean gestational age at the first ultrasound was 23±9 weeks, and the mean gestational age at the first antenatal visit was 24±11 weeks. After the COVID-19 pandemic, only a small proportion of mothers (3.2%) attended virtual clinics, and most of them (84.6%) attended only one clinic, with a higher mean gestational age of 30±7 weeks (Table 2).

**Table 2:** Frequency of antenatal parameters.

Parameter		Frequency	Percent
Follow-up	No	215	53.5%
	Yes	187	46.5%
Gravidity	1-2	197	49.0%
	3-4	128	31.8%
	≥5	77	19.2%
Parity	0	34	8.5%
	1-2	231	57.5%
	3-4	102	25.4%
	≥5	35	8.7%
Number of ultrasound examinations	0	145	36.1%
	1-2	235	58.5%
	≥3	22	5.5%
Gestational age in the first visit	Mean ± SD (range)	24±11 (2-42)	
Gestational age in the first ultrasound	Mean ± SD (range)	23±9 (5-41)	
Number of antenatal visits	0	86	21.4%
	1-	198	49.3%
	3-	70	17.4%
	≥5	27	6.7%
Did the patient attend a virtual clinic during COVID-19?	No	389	96.8%
	Yes	13	3.2%
Gestational age at the first virtual clinic (n=13)	Mean ± SD (range)	30±7 (18-40)	
Number of virtual clinics attended during COVID-19	1	11	84.6%
	2	2	15.4%

Association between antenatal parameters and follow-up

After examining the association between antenatal parameters and ultrasound follow-up, we found that having a prior cesarean section was positively associated with ultrasound attendance (p = 0.034). Additionally, having parity of one to three fetuses was significantly associated with ultrasound attendance (p = 0.013) (Table 3).

**Table 3:** Association between ultrasound and different antenatal parameters.

Parameter		Ultrasound		
		0	1-2	≥3
Mother’s age (years)	18-26	41 (36%)	64 (56.1%)	9 (7.9%)
	27-34	78 (41.3%)	102 (54%)	9 (4.8%)
	35-44	26 (26.3%)	69 (69.7%)	4 (4%)
	P-value	0.068		
Nationality	Non-Saudi	21 (42.9%)	26 (53.1%)	2 (4.1%)
	Saudi	124 (35.1%)	209 (59.2%)	20 (5.7%)
	P-value	0.552		
Prior cesarean section	No	106 (39.3%)	145 (53.7%)	19 (7%)
	Unknown	8 (40%)	12 (60%)	0 (0%)
	Yes	31 (27.7%)	78 (69.6%)	3 (2.7%)
	P-value	0.034		
Prior preterm	No	111 (34.8%)	189 (59.2%)	19 (6%)
	Unknown	22 (44.9%)	26 (53.1%)	1 (2%)
	Yes	12 (35.3%)	20 (58.8%)	2 (5.9%)
	P-value	0.611		
Follow-up	No	111 (51.6%)	101 (47%)	3 (1.4%)
	Yes	34 (18.2%)	134 (71.7%)	19 (10.2%)
	P-value	0.000		
Gravidity	1-2	70 (35.5%)	116 (58.9%)	11 (5.6%)
	3-4	46 (35.9%)	78 (60.9%)	4 (3.1%)
	≥5	29 (37.7%)	41 (53.2%)	7 (9.1%)
	P-value	0.447		
Parity	0	10 (29.4%)	19 (55.9%)	5 (14.7%)
	1-2	83 (35.9%)	140 (60.6%)	8 (3.5%)
	3-4	32 (31.4%)	62 (60.8%)	8 (7.8%)
	≥5	20 (57.1%)	14 (40%)	1 (2.9%)
	P-value	0.013		
Attending post-COVID-19 virtual clinic	No	141 (36.2%)	227 (58.4%)	21 (5.4%)
	Yes	4 (30.8%)	8 (61.5%)	1 (7.7%)
	P-value	0.884		

Regarding antenatal visits and virtual clinic attendance, we found that mothers who had a prior preterm delivery were significantly more likely to attend antenatal visits (p = 0.031), and this was also the case for mothers attending virtual clinics after COVID-19 (p < 0.001) (Table 4).

**Table 4:** Association between antenatal visits and different antenatal parameters.

Parameter		Antenatal visits			
		0	1-2	3-4	≥5
Mother’s age (years)	18-26	27 (24.5%)	56 (50.9%)	23 (20.9%)	4 (3.6%)
	27-34	40 (22.6%)	88 (49.7%)	33 (18.6%)	16 (9%)
	35-44	19 (20.2%)	54 (57.4%)	14 (14.9%)	7 (7.4%)
	P-value	0.545			
Nationality	Non-Saudi	17 (36.2%)	19 (40.4%)	7 (14.9%)	4 (8.5%)
	Saudi	69 (20.7%)	179 (53.6%)	63 (18.9%)	23 (6.9%)
	P-value	0.099			
Prior cesarean section	No	66 (25.8%)	129 (50.4%)	47 (18.4%)	14 (5.5%)
	Unknown	6 (31.6%)	10 (52.6%)	1 (5.3%)	2 (10.5%)
	Yes	14 (13.2%)	59 (55.7%)	22 (20.8%)	11 (10.4%)
	P-value	0.078			
Prior preterm	No	62 (20.7%)	165 (55.2%)	54 (18.1%)	18 (6%)
	Unknown	16 (33.3%)	21 (43.8%)	5 (10.4%)	6 (12.5%)
	Yes	8 (23.5%)	12 (35.3%)	11 (32.4%)	3 (8.8%)
	P-value	0.031			
Follow-up	No	84 (43.1%)	100 (51.3%)	6 (3.1%)	5 (2.6%)
	Yes	2 (1.1%)	98 (52.7%)	64 (34.4%)	22 (11.8%)
	P-value	<0.001			
Gravidity	1-2	43 (23%)	96 (51.3%)	37 (19.8%)	11 (5.9%)
	3-4	27 (22.3%)	66 (54.5%)	20 (16.5%)	8 (6.6%)
	≥5	16 (21.9%)	36 (49.3%)	13 (17.8%)	8 (11%)
	P-value	0.845			
Parity	0	6 (18.8%)	16 (50%)	9 (28.1%)	1 (3.1%)
	1-2	50 (22.9%)	122 (56%)	33 (15.1%)	13 (6%)
	3-4	19 (19%)	47 (47%)	22 (22%)	12 (12%)
	≥5	11 (35.5%)	13 (41.9%)	6 (19.4%)	1 (3.2%)
	P-value	0.136			
Attending post-COVID-19 virtual clinic	No	86 (23.4%)	195 (53%)	62 (16.8%)	25 (6.8%)
	Yes	0 (0%)	3 (23.1%)	8 (61.5%)	2 (15.4%)
	P-value	<0.001			

Outcomes for patients with appropriate care and lack of antenatal visits

We compared the outcomes for patients who received appropriate antenatal care and those who lacked antenatal visits. The group who received appropriate antenatal care had a significantly lower rate of medical complications during pregnancy (1.8%) compared to the group who lacked antenatal visits (7.4%) (p = 0.012). Additionally, the group who received appropriate antenatal care had a significantly lower rate of preterm delivery (7.2%) compared to the group who lacked antenatal visits (16.8%) (p = 0.016). However, the mean birth weight for the group who received appropriate antenatal care was 3.2±0.4 kg, while for those who lacked antenatal visits, it was 3.1±0.5 kg (p = 0.287).

## Discussion

In this study, we found that most mothers attended at least one antenatal visit or had a single ultrasound examination. However, a relatively large number still did not have either, which led us to examine the importance of both in the follow-up of patients.

Ultrasound specifically plays a vital role in diagnosing the viability of a fetus and is, therefore, accepted by the World Health Organization as a golden measure. The World Health Organization encourages performing an ultrasound scan before 24 weeks of gestation but not after that. Ultrasound scans can help detect anomalies and multiple pregnancies, improve a woman's pregnancy experience, and determine gestational age [10]. Although ultrasound scans are considered relatively safe during pregnancy, they can have psychological impacts [11]. For a prospective mother, attending a scan can be a happy experience, but it can also be a disturbing experience that increases anxiety levels through the detection of fetal abnormalities [12].

The relationship between ultrasound scans and cesarean sections should also be examined. In our study, we found a significant association between the two, which supports a previous study in China where researchers found that women with reported ultrasounds had a higher rate of cesarean sections [13]. The association can be attributed to several factors, including the anxiety that ultrasound scans might bring upon parents, especially mothers. Although ultrasound scans can result in feelings of satisfaction and happiness, they can also cause feelings of distress for the mother, which can increase her willingness to opt for a cesarean section. This distressful effect is not confined to mothers but also to physicians, as seeing a disturbed scan can cause worry and make them prefer cesarean section over natural delivery.

In our study, we found that 67.2% of women had prior cesarean section, which is a quite high percentage. This can be attributed to several factors previously mentioned in the literature. In Saudi Arabia, a study mentioned that the reasons mainly constituted easy access to anesthesia and neonatal care units, involvement of juniors in decision making, and inconsistency in the antenatal care provided [14]. It can also be the ingrained thought that cesarean section is safer and is associated with a lower mortality rate. This hypothesis is supported by a study conducted in the United States, which found that cesarean section provides a better survival advantage for infants between 22 and 25 weeks [15]. However, cesarean section is not risk-free, and it has some serious drawbacks and disadvantages that may threaten the life of the mother and the fetus. These disadvantages include infection, hemorrhage, bladder or bowel injury, embolism, clots, or injury to the baby [16]. Thus, it is advised that unless there is a medical indication for cesarean section, there should be no need to perform it. Therefore, the government should take serious steps to reduce the number of unnecessary cesarean section surgeries.

In our study, we found a significant association between prior preterm delivery and antenatal visits. This significant association can be explained in terms of the worry mothers face after encountering an incident, such as having a preterm delivery, so they take further precautions in the following pregnancies. This can be used to promote antenatal visits among new mothers without spreading any unnecessary fear. Additionally, we cannot tell for sure why the association was present in the case of antenatal visits but not in the case of the ultrasound scan, but we can hypothesize that there is a lack of awareness in terms of ultrasound scans despite their being more accurate.

We also noted a significant association between parity and ultrasound scans. This indicates that women are more likely to go for an ultrasound if they have more children. This was also the case in a previous Nigerian study where parity significantly influenced women's decision to undergo ultrasound examination [9]. One possible explanation for this association is that women who have had more children may be more experienced with pregnancy and childbirth and therefore may be more aware of the potential benefits of ultrasound scans. Additionally, these women may be more likely to have had previous experiences with complications during pregnancy or childbirth, which may have increased their motivation to undergo ultrasound scans. However, further research is needed to better understand the reasons behind this association and to develop effective strategies to promote ultrasound scans among all pregnant women, regardless of parity.

Our study found that primigravida women had reduced compliance with antenatal care, which can be attributed to several reasons. Firstly, primigravida women may lack prior experience with pregnancy and may have limited knowledge and understanding of the importance of antenatal care. Additionally, socio-economic factors such as limited financial resources, lack of social support, and difficulty accessing healthcare services could also contribute to reduced compliance. Cultural beliefs and perceptions about pregnancy and antenatal care may also play a role, as some cultures view pregnancy as a natural process that does not require medical intervention. Finally, healthcare providers' attitudes and communication skills could impact compliance, as some women may feel uncomfortable or intimidated during healthcare interactions.

Retrospective studies have inherent limitations, such as the inability to control for confounding variables and the reliance on previously recorded data. As such, this study is limited in its ability to establish causality between antenatal care during the COVID-19 pandemic. Additionally, the study was conducted at a single center, which may limit the generalizability of the findings to other settings. The patient population and healthcare system in Jeddah, Saudi Arabia may differ from those in other regions or countries, and the impact of the COVID-19 pandemic on antenatal care may vary accordingly. Finally, there may have been selection bias in this study. Patients who did not attend antenatal care at the hospital or had incomplete medical records were excluded from the study, which may have resulted in a biased sample. Additionally, patients who attended virtual clinics may differ systematically from those who did not attend, which may confound the relationship between virtual clinic attendance and delivery outcomes. Despite these limitations, this study provides valuable insights into the impact of the COVID-19 pandemic on antenatal care in a specific population. The findings may be useful for healthcare providers and policymakers in developing strategies to optimize antenatal care during similar public health crises in the future.

## Conclusions

Our study revealed that there was only moderate adherence to antenatal care procedures and that expectant mothers underwent cesarean sections more frequently than natural deliveries. These findings highlight the need for improvements in antenatal care procedures and greater efforts to promote natural delivery. We strongly recommend that authorities prioritize the implementation of campaigns and initiatives aimed at educating and supporting expectant mothers on the benefits of antenatal care and natural delivery. By doing so, we can enhance maternal and infant health outcomes and ensure that every mother and child receives the best possible care.
